# *Rattus* population genomics across the Haida Gwaii archipelago provides a framework for guiding invasive species management

**DOI:** 10.1111/eva.12907

**Published:** 2020-01-08

**Authors:** Bryson M. F. Sjodin, Robyn L. Irvine, Adam T. Ford, Gregg R. Howald, Michael A. Russello

**Affiliations:** ^1^ Department of Biology University of British Columbia Kelowna BC Canada; ^2^ Gwaii Haanas National Park Reserve National Marine Conservation Area Reserve and Haida Heritage Site Skidegate BC Canada; ^3^ Island Conservation Santa Cruz California USA

**Keywords:** conservation, invasive species, island biogeography, population genetics, *Rattus norvegicus*, *Rattus rattus*

## Abstract

Invasive species have led to precipitous declines in biodiversity, especially in island systems. Brown (*Rattus norvegicus*) and black rats (*R. rattus*) are among the most invasive animals on the planet, with eradication being the primary tool for established island populations. The need for increased research for defining eradication units and monitoring outcomes has been highlighted as a means to maximize success. Haida Gwaii is an archipelago ~100 km off the northern coast of British Columbia, Canada, that hosts globally significant breeding populations of seabirds that are at risk due to invasive rats. Here, we paired sampling of brown (*n* = 287) and black (*n* = 291) rats across the Haida Gwaii archipelago with genotyping by sequencing (10,770–27,686 SNPs) to investigate patterns of population connectivity and infer levels/direction of gene flow among invasive rat populations in Haida Gwaii. We reconstructed three regional clusters for both species (north, central and south), with proximate populations within regions being largely more related than those that were more distant, consistent with predictions from island biogeography theory. Population assignment of recently detected individuals post‐eradication on Faraday, Murchison and the Bischof Islands revealed all were re‐invaders from Lyell Island, rather than being on‐island survivors. Based on these results, we identified six eradication units constituting single or clusters of islands that would limit the potential for reinvasion, some of which will need to be combined with biosecurity measures. Overall, our results highlight the importance of targeted research prior to conducting eradications and demonstrate a framework for applying population genomics for guiding invasive species management in island systems.

## 1 INTRODUCTION

Understanding dispersal patterns, population connectivity and subsequent gene flow of invasive species provides critical information for developing management strategies to reduce or ameliorate their impacts on biodiversity and ecosystem services (Abdelkrim, Pascal, Calmet, & Samadi, 2005). Yet it can be difficult to measure levels of dispersal and gene flow by traditional visual‐ or telemetry‐based methods. Invasive rats in island systems provide one such example; it is near impossible to monitor intervening ocean passages for individuals swimming from island to island (Russell & Clout, 2004). Also, rats that disperse via human‐mediated means (*e.g.* boats) are often cryptic. Even if dispersers are detected, the origin of these individuals may remain unknown if the vessel docked at multiple ports or adjacent to rat‐infested islands en route. Additionally, fine‐scale environmental data that influence dispersal (such as velocity of ocean currents through a passage) may simply not exist, and even well‐documented natural barriers to gene flow may be rendered insignificant due to the commensal spread of invasive rats.

Population genetics and genomics can be used to infer gene flow to inform invasive species management, without the need to track the movement of individual dispersers. For example, Abdelkrim et al. (2005) genotyped brown rats (*Rattus norvegicus*; also known as Norway rats) across numerous islands located off the coast of Brittany, France, at eight microsatellites, revealing extensive gene flow to the degree that islands within a few hundred metres of each other should be considered a single population. Robertson and Gemmell (2004) coined these connected populations as an “eradication unit,” recommending that all populations within the unit be eradicated simultaneously to prevent failure by reinvasion. Such investigations are important, as complete eradication of rats can prove to be challenging due to their high adaptability and fecundity (Holmes et al., 2015; Howald et al., 2007; Russell et al., 2010; Simberloff, 2003).

On islands throughout the world, rodent eradication attempts have resulted in a ~10% failure rate (Howald et al., 2007); identifying the cause of eradication failure is of paramount importance given the financial, logistical and social costs of additional management actions. There are two prevailing hypotheses associated with rodent eradication failure including the following: (a) survivor hypothesis and (b) re‐invader hypothesis. The survivor hypothesis argues that eradications failed because of inadequate distribution or toxicity of rodenticide designed to kill rats. Bait containing rodenticide is distributed either by broadcasting by hand or helicopter, or by deployment of bait stations in strategic and systematic locations in every potential rat territory (Howald et al., 2007). If the poison bait is inadequately spread throughout the population's distribution, some individuals may never encounter the bait or rodenticide and thus survive the attempt. Alternatively, a poison bait regime may fail due to insufficient toxicity or a development of resistance in the invasive population (Amos, Nichols, Churchyard, & Brooke, 2016; Holmes et al., 2015). In both cases, this could result in a small handful of individuals surviving the eradication process to re‐establish the invasive population.

The re‐invader hypothesis argues that eradications fail due to dispersal from a source population. This scenario can be particularly difficult to investigate without sufficient pre‐ and post‐eradication genetic samples to adequately capture the extent of genetic variation in either population (Abdelkrim, Pascal, & Samadi, 2007; Howald et al., 2007; Savidge et al., 2012). Often, it is the lack of pre‐eradication samples that acts as a barrier to effectively elucidate the origin of a population, as these are necessary to properly characterize the historical genetic composition (Abdelkrim et al., 2005, 2007).

Both brown and black (*R. rattus*) rats have invaded Haida Gwaii (X̱aayda Gwaay in Haida), an archipelago ~100 km off the northern coast of British Columbia often likened to Canada's version of the Galápagos Islands due to high levels of unique biodiversity, rare species and endemism (Calder, Taylor, & Mulligan, 1968; Foster, 1965; Moodie & Reimchen, 1973; for reviews see Gaston, Golumbia, Martin, & Sharpe, 2008; Golumbia, 1999). Consisting of two large main islands and ~150 smaller islands, Haida Gwaii also represents a significant breeding site for 1.5 million seabirds across twelve species, in some cases, representing large proportions of the total species’ population (Harfenist, 2003). Brown rats arrived sometime in the late 1800s to early 1900s based on local accounts, though the first confirmed naturalist record did not occur until 1981 (Bertram & Nagorsen, 1995; Gaston et al., 2008; Golumbia, 1999). Black rats are thought to have first invaded Haida Gwaii from European ships in the 1700s with the first recorded occurrence in 1908 (Gaston et al., 2008; Harrison, 1925). Since their arrival in Haida Gwaii, rats of both species have had devastating impacts on native sea birds, including significant population declines for the ancient murrelet (*Synthliboramphus antiquus*), Cassin's auklet (*Ptychoramphus aleuticus*), fork‐tailed storm petrel (*Oceanodroma furcate*), Leach's storm petrel (*O. leucorhoa*), rhinoceros auklet (*Cerorhinca monocerata*) and tufted puffin (*Fratercula cirrhata*) (Gaston et al., 2008; Harfenist, 2003).

To meet management objectives of both protecting ecological and cultural integrity (Parks Canada Agency, 2018), Parks Canada and other agencies are taking steps to eliminate the negative effects of invasive rats in Haida Gwaii. Eradication has been the primary tool used to date, as management through population suppression (*e.g.* using poison regimes aimed at reduction rather than removal) is ineffective in species with high fecundity, density‐dependent reproduction and the capacity to adapt through bait resistance or neophobia (Damin‐Pernik et al., 2017; Emlen, Stokes, & Winsor, 1948; Takács, Kowalski, & Gries, 2016; Zipkin, Kraft, Cooch, & Sullivan, 2009). Both species of rats were successfully eradicated from the northern islands of Langara, Lucy and Cox (1997); the southern St. James Island (1998); and the east‐central island of Arichika (2011) (Gaston et al., 2008; Gill, Wein, Howald, & McClelland, 2014; Golumbia, 1999; Kaiser et al., 1997). Black rats were successfully eradicated from Faraday Island and Murchison Island (2013), but they have both since been invaded by brown rats. Similarly, brown rats have invaded House and Hotspring Islands, which now threatens Ramsay Island, host to the most significant seabird colonies in Gwaii Haanas National Park (Harfenist, 2003). Two eradications have been attempted on the Bischof Islands (2003, 2011), a small group of islets on the east‐central coast of Haida Gwaii; however, there have been subsequent detections of brown rats via camera traps post‐eradication. In all cases, the source(s) of the current invasive populations are unknown.

Here, we paired archipelago‐wide sampling of brown and black rats with genotyping by sequencing to investigate patterns of population connectivity and infer levels/direction of gene flow among invasive rat populations in Haida Gwaii. We used this information to identify candidate islands and define eradication units that present the lowest risk of reinvasion. Lastly, we investigated the source(s) of recent invasions, including an explicit testing of the survivor and re‐invader hypotheses in the Bischofs, to evaluate existing eradication methodology and inform biosecurity measures.

## 2 MATERIALS AND METHODS

### 2.1 Study site and sample collection

**Figure 1:**
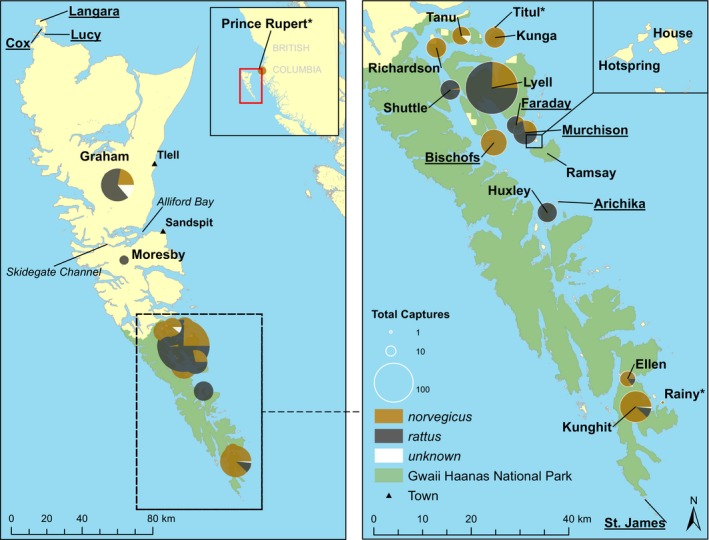
Distribution and sample size of brown (*Rattus norvegicus*) and black rats (*R. rattus*) collected in Haida Gwaii, BC. Rats were collected from 2008 to 2018 by Parks Canada staff. (*) indicates a single capture. Underlined island names indicate the locations of past eradications

All sample collection was performed by Parks Canada staff from 2008 to 2018. Based on qualitative morphological assessment in the field, 287 putative brown rats, 296 putative black rats and 15 morphologically ambiguous rats were sampled across 12 islands throughout Haida Gwaii (Figure 1; see Tables 1 and 2 for Haida island names). One additional brown rat sample was collected from Prince Rupert, the closest port on mainland BC. Sampling was focused on the Gwaii Haanas National Park Reserve, National Marine Conservation Area Reserve and Haida Heritage Site (hereafter, Gwaii Haanas), as it represents an important site both culturally and ecologically; approximately half of the 1.5 million seabirds in Haida Gwaii are found in Gwaii Haanas as well as several historical Haida village sites (Parks Canada Agency, 2019a, 2019b). Most individuals were live trapped using Tomahawk collapsible traps, with some individuals being collected during island‐wide carcass searches following an eradication attempt. Sample locations were also chosen to be proximate to nesting seabird habitat. Traps were deployed along the shoreline at approximately 30‐m intervals above the high‐tide mark and partially concealed with moss and bark to prevent tampering by predatory birds (*e.g.* ravens, eagles) and to keep rats dry once captured. As rats are primarily nocturnal, the traps were set in the late afternoon or early evening and baited with a combination of canned sardines and the commercially available rodent attractant Provoke (Bell Laboratories, Inc.), and checked the following morning. This process was repeated for up to 3 days for each sampling excursion at each location. Upon successful capture, rats <250 g were first anesthetized using isoflurane and then euthanized via cervical dislocation; rats >250 g were euthanized with a strong dose of isoflurane. Whole ears were removed for downstream genomic analysis. Sample collection was performed under Parks Canada Agency Animal Care Committee protocol GHNPR11‐5.

**Table 1:** Sample size and genetic diversity estimates for 12 brown rat (*Rattus norvegicus*) populations in Haida Gwaii, BC

Population	*N*	*N*_A_	*N*_E_	*H*_o_	*H*_s_	*G*_is_
Post‐Bischofs (Kingts'ii Gwaay.yaay)	21	1.580	1.280	0.178	0.174	−0.020
Pre‐Bischofs	28	1.464	1.119	0.123	0.123	−0.000
Ellen (Kilgii Gwaay)	19	1.652	1.353	0.211	0.215	0.019
Faraday (K’aaxada Gwaay)	6	1.325	1.222	0.176	0.134	−0.308
Tlell^a^ (Tll.aal)	14	1.489	1.106	0.049	0.088	0.439
NW‐Kunghit (Gangxid Gwaay.yaay)	36	1.820	1.432	0.266	0.259	−0.027
E‐Kunghit^b^	27	1.720	1.418	0.254	0.248	−0.028
Kunga^c^ (K’ang.guu Gwaay.yaay)	32	1.541	1.275	0.170	0.165	−0.028
Lyell (Hlgaa Gwaay)	34	1.727	1.321	0.197	0.202	0.027
Murchison (Gaysiigas Gwaay)	10	1.391	1.243	0.164	0.149	−0.097
Richardson (Sgaanagwaay Gwaay.yaay)	30	1.720	1.354	0.232	0.217	−0.071
Tanu (T’aanuu Gwaay)	26	1.680	1.367	0.226	0.223	−0.010
Total	283	2.000	1.233	0.187	0.184	−0.020

Abbreviations: *G*_is_, inbreeding coefficient; *H*_o_, mean observed heterozygosity; *H*_s_, mean heterozygosity within population; *N*, number of individuals; *N*_A_, mean number of alleles per locus; *N*_E_, mean number of effective alleles per locus.

Based on results from the clustering analyses:

a Tlell (Graham Island) population also includes *n* = 1 rat from Prince Rupert, BC.

b E‐Kunghit Island also includes *n* = 1 rat from Rainy Islands.

c The Kunga Island population also includes *n* = 1 rat from Titul Island (Taadlls Gwaay.yaay).

Haida names for the islands are indicated in parentheses.

**Table 2:** Sample size and genetic diversity estimates for 12 black rat (*Rattus rattus*) populations in Haida Gwaii, BC. Haida names for the islands are indicated in parentheses

Population	*N*	*N*_A_	*N*_E_	*H*_o_	*H*_s_	*G*_is_
Faraday (K’aaxada Gwaay)	14	1.490	1.278	0.160	0.170	0.059
S‐Graham (T’aaxwii Xaaydaga Gwaay.yaay iinagwaay)	23	1.675	1.361	0.194	0.217	0.107
N‐Graham	24	1.606	1.318	0.173	0.189	0.083
Huxley (Gaaduu Gwaay)	15	1.538	1.272	0.169	0.168	−0.004
Kunghit (Gangxid Gwaay.yaay)	4	1.380	1.216	0.147	0.152	0.036
Lyell‐FP (Hlgaa Gwaay)	51	1.700	1.296	0.185	0.181	−0.021
Lyell‐SW	56	1.763	1.321	0.199	0.199	−0.003
Murchison (Gaysiigas Gwaay)	22	1.665	1.349	0.206	0.213	0.030
Sandspit (K’il Kun Llnagaay)	5	1.417	1.246	0.182	0.164	−0.110
Shuttle (Gwaay Daagaaw)	24	1.549	1.279	0.171	0.169	−0.013
Total	238	2.000	1.224	0.179	0.183	0.022

Abbreviations: *G*_is_, inbreeding coefficient; *H*_o_, mean observed heterozygosity; *H*_s_, mean heterozygosity within population; *N*, number of individuals; *N*_A_, mean number of alleles per locus; *N*_E_, mean number of effective alleles per locus.

### 2.2 DNA extraction and library preparation

Whole genomic DNA (gDNA) was extracted from 10 to 20 mg of dried ear tissue using the Qiagen DNeasy^®^ Blood and Tissue Kit and treated with RNase A (5PRIME) following the manufacturer's protocol. Double‐digest restriction enzyme‐associated DNA sequencing (ddRAD) libraries were constructed using a modified protocol described by Puckett et al. (2016; see also Peterson, Weber, Kay, Fisher, & Hoekstra, 2012). Approximately 1µg of gDNA was digested from each individual using the restriction enzymes MluCI and SphI‐HF (New England Biolabs^®^ Inc.), and a unique combinatorial barcode and index (New England Biolabs^®^ Inc) was ligated onto the 5′ and 3′ ends of the resultant fragments, respectively. Barcoded individuals were pooled in equimolar concentrations into libraries (*n = *96 individuals/library). Approximately 400bp fragments were size‐selected using a Pippin Prep™ (Sage Science), and the size‐selected libraries were PCR amplified for 12 cycles using Phusion PCR reagents (New England Biolabs^®^ Inc). In total, seven libraries were constructed, with samples replicated within (*n* = 11) and among (*n* = 13) libraries to evaluate genotyping error rates. Libraries were sequenced using one full lane of Illumina HiSeq 2500 PE125 (125bp, paired‐end) per library.

### 2.3 Demultiplexing and species determination

Raw sequence reads were demultiplexed to individuals using the *process*_*radtags* command in stacks v2.0 (Catchen, Hohenlohe, Bassham, Amores, & Cresko, 2013); during this process, barcodes and indices were removed, and the reads trimmed to 100 bp to remove low‐quality bases at the 3′ ends. Processed reads were then aligned to the brown rat reference genome (Rnor_6.0, GenBank assembly accession: GCA_000001895.4) using the software Bowtie 2 v2.2.9 (Langmead & Salzberg, 2012). A minimum of 40% alignment to the reference genome was employed as a form of quality control, and individuals failing to meet this threshold (*e.g.* due to poor sequence quality, failed adapter ligation or DNA amplification from a nontarget species such as bacteria) were removed from downstream analysis.

Putative SNP loci were first identified and genotyped across all individuals using the *gstacks* and *populations* programs in stacks v2.0 (Catchen et al., 2013) requiring a locus to be genotyped in at least 90% of the individuals (*r* = .90) and have a minor allele frequency exceeding 5% (min_maf = 0.05). This initial data set was used to designate individuals to species, as morphological identification can be difficult with juveniles as well as for some phenotypes. To assign individuals to species, we used a discriminant analysis of principle components as implemented by the R‐package *adegenet* v2.1.1 (Jombart & Ahmed, 2011), which first applies principle component analysis to identify genetic clusters and then uses discriminant analysis to maximize the variation among these clusters while minimizing within‐cluster variation (Jombart, Devillard, & Balloux, 2010). Individuals were separated into species‐specific data sets; samples with <90% assignment to either species or inconsistent species assignment between replicates were removed from downstream analyses.

### 2.4 SNP genotyping and filtering

Each species‐specific data set was independently run through the *gstacks* program in stacks. Due to the relatively high frequency of sequencing errors associated with the Illumina platform (see Pfeiffer et al., 2018), a sensitivity analysis was performed to ensure robust and accurate genotypes through the *populations* program in stacks. We varied the proportion of genotyped individuals (‐r) to call a SNP from 0.70 to 0.95 and the minimum minor allele frequency (‐‐min_maf) from 0.01 to 0.05 to identify optimum parameters. Only a single SNP per RADtag was retained (‐‐write_single_snp) to minimize the potential for linkage disequilibrium, and the maximum observed heterozygosity (‐‐max_obs_het) was set to 0.50 for all filtering iterations. Individual mean depth, missing data per individual, number of SNPs and number of individuals with ≥6× depth of coverage were calculated for each filtered data set using VCFtools v0.1.15 (Danecek et al., 2011). Once final filtering parameters were chosen, low coverage (<6×) individuals were removed, and each data set was passed a final time through *populations* using the identified optimal filtering parameters. SNPs located on the X‐chromosome were removed using PLINK v1.90b5 (Purcell et al., 2007). *F*_ST_ outliers were detected using the method of Beaumont and Balding (2004) as implemented in BayeScan v2.1 (Foll & Gaggiotti, 2008) using 100,000 iterations with a 50,000 iteration burn‐in and a prior odds value of 10. Outlier loci were defined as having a mean *q*‐value of 0.20 over five runs and were removed from downstream analysis. Each data set was split into putative populations structured by island, and then, all loci were assessed for significant (α = .05) deviation from Hardy–Weinberg equilibrium (HWE) using VCFtools v0.1.15 (Danecek et al., 2011). A locus was removed if it significantly deviated from HWE in at least 50% of populations with a minimum of *n* = 2 individuals.

Genotyping error rate was estimated by calculating the rate of discordance among replicate samples both within and across sequencing libraries. Standard measures of genetic diversity, including mean number of alleles per locus, observed/expected heterozygosity and inbreeding coefficients, were assessed across populations within each data set using GenoDive v2.0b27 (Meirmans & Tienderen, 2004).

### 2.5 Assessment of population structure

Several approaches were used to detect population structure. First, we used PCA to infer genetic clusters within each species and across all populations using the R‐package *SNPRelate* v1.16.0 (Zheng et al., 2012). We then ran independent PCA on regional clusters of islands to detect finer scale structure. We estimated population differentiation among identified clusters by calculating *θ* (Weir & Cockerham, 1984) for all pairwise observations using 1,000 permutations as implemented in Genetix v4.05.2 (Belkhir, Borsa, Chikhi, Raufaste, & Bonhomme, 2004). For larger island populations, we also estimated pairwise *θ* among all within‐island sample sites; if no differentiation was detected, all sample sites within the island were considered a single population. We estimated admixture coefficients using the *snmf()* function in the R‐package *LEA* v2.6.0 (Frichot & François, 2015). This function uses sparse non‐negative matrix factorization to estimate individual ancestry coefficients from a genotypic matrix. Additionally, the analysis is robust to deviation from Hardy–Weinberg equilibrium as well as unequal sample sizes among groups. For this analysis, we examined *k* = 1–20 over 10 iterations of each *k*. We plotted the cross‐entropy criterion for each *k* and identified the “elbow” as the optimal number of clusters as recommended (Frichot & François, 2015). All runs for the optimal *k* were summarized using CLUMPP v1.1.2 (Jakobsson & Rosenberg, 2007) and visualized using the R‐package *pophelper* v2.2.3 (Francis, 2017).

To examine whether the interior of Kunghit Island was acting as a barrier to dispersal, we calculated the shortest distance between each pair of populations along the shoreline (hereafter, shoreline distance), allowing for movement up to 1 km from the coastline, as this has been previously proposed to be the maximum extent of rat movement inwards on islands (Harper, 2006; Pye & Bonner, 1980; Pye, Swain, & Seppelt, 1999). We tested a hypothesis of isolation by distance (IBD) using Mantel tests where Euclidean distance and shoreline distance were considered separately as predictors of genetic distance (measured as *θ*/1‐*θ*).

### 2.6 Directional migration rates among populations

We evaluated rates of recent migration among populations using a Bayesian framework as implemented by the software BA3‐SNPs v1.1.0 (Mussmann, Douglas, Chafin, & Douglas, 2019; Wilson & Rannala, 2003). This analysis uses multilocus genotypes to infer recent gene flow and estimates both the magnitude and the direction of migration. For each species, we used 10 million iterations and a burn‐in period of 1 million steps, sampling at 100 iteration intervals. The migration rates, allele frequencies and inbreeding coefficient mixing parameters were adjusted to achieve acceptance rates between 0.2 and 0.6 as recommended by the user manual. This analysis was completed five times for each species, with each run initialized by a different random seed, to assess chain convergence. We constructed 95% credible sets using the mean migration rate across runs minus 1.96 times the mean standard deviation as recommended (Wilson & Rannala, 2003). Migration rates were considered significant if the credible set did not include zero.

### 2.7 Identifying source population(s) for the Bischof Islands and novel invasions

A series of population assignment tests were used to identify the source population for the failed eradication on the Bischof Islands as well as the recent brown rat invasions on Faraday Island and Murchison Island. We used a projected PCA using the *smartpca* function in the software package EIGENSOFT v7.2.1 (Galinsky et al., 2016; Patterson, Price, & Reich, 2006; Price et al., 2006), which first defines the parameter space using only the reference samples, then projects samples of unknown origin onto this space. For the Bischof Islands, we used Lyell Island and Richardson Island as putative source populations, as these are the most proximate islands with brown rat populations (*N.B.* Faraday Island and Island Murchison were invaded after the Bischofs population re‐appeared). Additionally, the pre‐eradication population was considered as a reference population under the survivor hypothesis. For the Faraday and Murchison Island invasions, Lyell Island, Richardson Island, the post‐eradication population in the Bischof Islands and the town of Tlell, BC (Graham Island), were examined as the putative source. These populations were again chosen for their proximity, except in the case of Tlell, which was considered because recent lumber shipments from Tlell to Faraday Island posed a potential introduction pathway. We also assigned individuals to populations following the method outlined by Rannala and Mountain (1997) as implemented in GeneClass2.0 (Piry et al., 2004), which looks to detect immigration by estimating population allele frequencies using Bayesian methods, then calculates the probability of a genotype arising from each defined population. Due to computation limitations associated with GeneClass2.0, these analyses were based on a random subset of 5,000 SNPs selected using the ‐‐thin‐count option in PLINK (Purcell et al., 2007).

## 3 RESULTS

### 3.1 Species determination and data set quality

DNA sequencing resulted in approximately 215–260 million high‐quality reads per library (Table S1). Following demultiplexing and reference alignment, we identified 287 unique brown rats and 291 unique black rats based on the genetic analysis; 20 unique individuals were ambiguous or inconsistent among replicates in their species assignment and were removed from downstream analyses (Figure S1). For the brown rats, we retained loci genotyped in at least 80% of individuals and having a minimum minor allele frequency of 5% based on the results of the sensitivity analysis, parameters that were also consistent with a previous brown rat population genomic study (Puckett et al., 2016); the locus and individual filtering resulted in 27,686 SNPs across 283 unique individuals (*n* = 297 with replicates; Table S2). We initially chose the same filtering parameters for the black rats as the brown rats; however, genotyping error between replicate samples was high (>13%; see Table S3 for sensitivity analysis). To compensate, we retained loci genotyped in at least 95% of individuals and with a minimum minor allele frequency of 5%; the locus and individual filtering resulted in 10,770 SNPs across 238 unique individuals (*n* = 242 with replicates). Mean within‐ and among‐library genotyping error rates were 2.0% and 2.6%, respectively, for the brown rats and 4.2% and 2.5% for the black rats (Table S4).

### 3.2 Genetic diversity, population differentiation and migration: brown rats

Genetic diversity was high across all brown rat populations except for the Tlell (Graham Island) population, which had relatively lower levels of heterozygosity and effective number of alleles, and higher levels of inbreeding (Table 1). We detected low, but significant levels of differentiation among proximate brown rat populations (Table S5). The Tlell (Graham Island) population was highly divergent from all populations (pairwise *θ* > 0.55 for all comparisons). Differentiation among sample sites on Lyell Island was low (pairwise *θ* < 0.07 for all comparisons); as such, all were grouped into a single Lyell population. Brown rats sampled from Arnold Point, Bowles Point and Gilbert Bay on Kunghit Island were not significantly differentiated, and rats from Hornby Point displayed low, but significant differentiation from these sites (pairwise *θ* < 0.06 for all comparisons); consequently, these sites were grouped into a single “NW‐Kunghit” population. There was significant, albeit low, differentiation among the east Kunghit sample sites of Marshall Island and Keeweenah Bay; as such, these sites were grouped into a single “E‐Kunghit” population. The Luxana Bay population displayed low‐to‐moderate differentiation from both the NW‐ and E‐Kunghit populations and was grouped with the E‐Kunghit population due to geography; additionally, the Rainy Island population was not significantly differentiated from any of the Kunghit sites and was also grouped with the E‐Kunghit population due to geography.

We found significant isolation‐by‐distance patterns using both Euclidean distance (Mantel's *r* = .790; *p* < .001) and the shoreline distance (Mantel's *r* = .802; *p* < .01) among Kunghit Island sample sites. Shoreline distance was more strongly correlated with genetic distance than Euclidean distance, though the difference was marginal.

**Figure 2:**
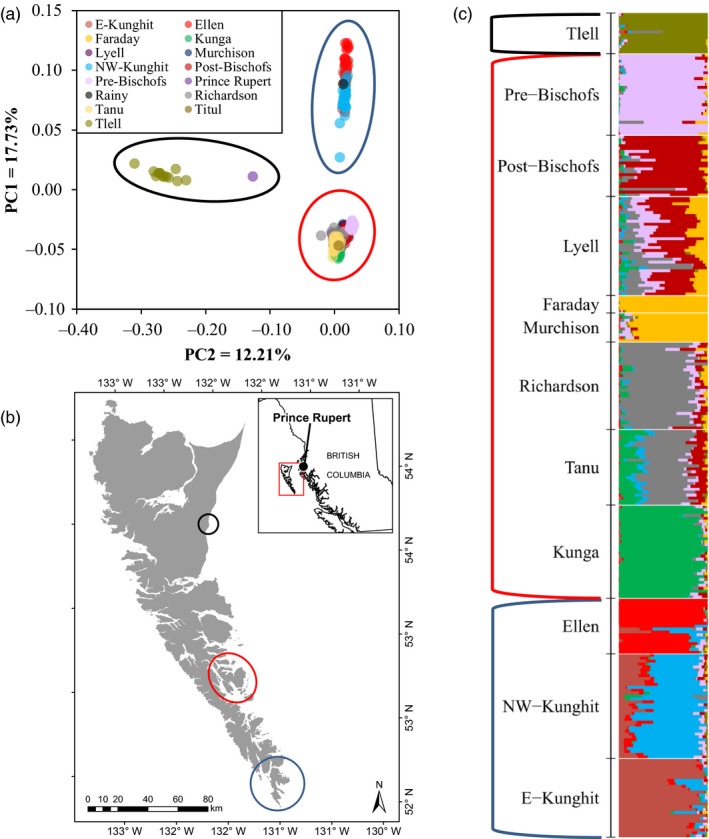
(a) Principle component analysis (PCA) and (c) ancestry coefficients for *n* = 283 brown rats (*Rattus norvegicus*) collected in Haida Gwaii, BC. PCA was performed using the R‐package *SNPRelate* v1.16.0 (Zheng et al., 2012), and ancestry coefficients (*k* = 9) were estimated using sparse non‐negative matrix factorization implemented by the R‐package *LEA* v2.4.0 (Frichot & François, 2015). Three regionally distinct clusters were identified indicated by the coloured ovals on (a) and (b) and by coloured brackets on (c). The single sample from Titul Island was included in the “Kunga” label, and the single sample from the Rainy Islands was included in the “E‐Kunghit” label to increase readability

We detected three distinct clusters in the brown rats using PCA, which segregated based on geography (Figure 2a,b). Central populations formed a discrete cluster, as did southern populations, and rats collected from Tlell (Graham Island) formed their own unique cluster. The single mainland sample from Prince Rupert did not cluster with any region. PCA of the central cluster indicated some substructure among islands (Figure S2A). Lyell and the post‐eradication population on the Bischof Islands formed a single cluster, as did Faraday and Murchison. Tanu and Richardson appeared to share some common ancestry, but still formed discrete clusters. Both the Kunga population and the pre‐eradication population on the Bischof Islands appeared to be quite divergent from all other populations. We further detected substructure among the southern cluster (Figure S2B). We detected some divergence among north‐west and eastern sites on Kunghit Island. Ellen also appeared to be divergent from Kunghit Island.

We found an optimal number of genetic clusters *k* = 9 for the brown rats based on results from *LEA* (Figure S3). The brown rats clustered by island, with Faraday and Murchison representing a single unit, as did Tanu and Richardson (Figure 2c). We also found the NW‐ and E‐Kunghit populations formed largely discrete clusters with some mixed ancestry (Figure S4). The Lyell Island population displayed substantial coancestry with several proximate populations. There was no evidence of mixed ancestry between the pre‐ and post‐eradication populations on the Bischof Islands.

We detected significant migration rates from the post‐eradication population on the Bischof Islands to Lyell Island, as well as from the E‐Kunghit to NW‐Kunghit Island populations (Table S6). Additionally, we identified *n* = 4 first‐generation migrants consistently across runs, which were congruent with the clustering‐based approaches. The single Rainy Islands individual was identified as a first‐generation migrant from the E‐Kunghit Island population and supports a single genetic cluster between these two groups. Furthermore, the single Titul Island individual was identified as a first‐generation migrant from Kunga Island, also supporting a single genetic cluster between these two islands. All other migration rates were not significant.

### 3.3 Genetic diversity, population differentiation and migration: black rats

Genetic diversity across black rat populations was also high, though Graham Island did show low levels of inbreeding (Table 2). Overall, pairwise differentiation among black rat populations was relatively higher than among brown rat populations (Table S7). Proximate populations showed low‐to‐moderate population differentiation. The Graham Island sample sites grouped into northern and southern populations and were moderately differentiated (pairwise *θ* = 0.142). There was low‐moderate differentiation (pairwise *θ* = 0.07–0.12) between the Faraday Passage and all other Lyell Island populations, but low differentiation (pairwise *θ* < 0.06) among the remaining Lyell sample sites.

**Figure 3:**
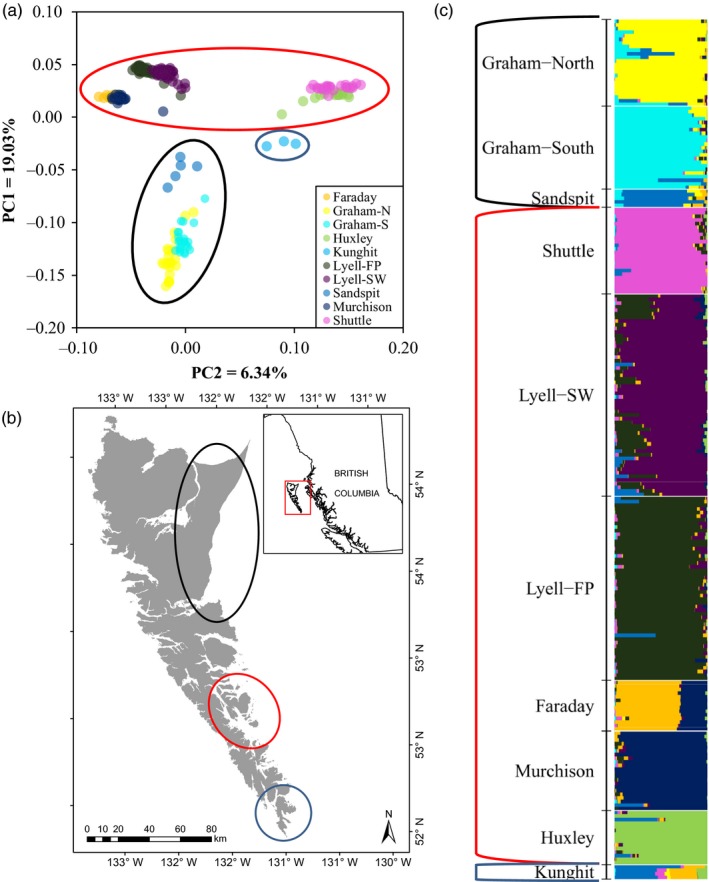
(a) Principle component analysis (PCA) and (c) ancestry coefficients for *n* = 238 black rats (*Rattus rattus*) collected in Haida Gwaii, BC. PCA was performed using the R‐package *SNPRelate* v1.16.0 (Zheng et al., 2012), and ancestry coefficients (*k* = 9) were estimated using sparse non‐negative matrix factorization implemented by the R‐package *LEA* v2.4.0 (Frichot & François, 2015). Three regionally distinct clusters were identified indicated by the coloured ovals on (a) and (b) and by coloured brackets on (c)

We found similar regional clustering among black rat populations as we did with the brown rats; however, each cluster was less discrete than the three brown rat clusters (Figure 3a,b). The northern cluster consisted of populations on Graham Island and Sandspit, BC, on Moresby Island. We detected divergence among northern and southern sample sites within Graham Island, as well as separation between Graham Island and Sandspit (Figure S5A). We found three subclusters within the central group: Faraday Island and Murchison Island; Shuttle Island and Huxley Island; and the two Lyell Island populations (Figure S5B). There was some divergence seen between the Faraday Passage (“Lyell‐FP”) and southwest Lyell (“Lyell‐SW”) populations, which corresponded well with the above pairwise *θ* estimates (Table S7).

We found an optimal *k* = 9 genetic clusters for the black rats (Figure S6). As with the brown rats, these genetic clusters largely segregated by island (Figure 3c; Figure S7). The north and south Graham Island populations each represented a unique cluster with some mixed ancestry between them. The “Lyell‐SW” and “Lyell‐FP” subpopulations formed discrete clusters, indicating substructure within Lyell Island.

We did not detect significant migration among black rat populations except for two populations on Lyell Island; we detected significant migration from the “Lyell‐SW” to the “Lyell‐FP” subpopulations (Table S8). We did not detect any first‐generation migrants for any island pairs.

### 3.4 Source(s) of novel brown rat invasions

**Figure 4:**
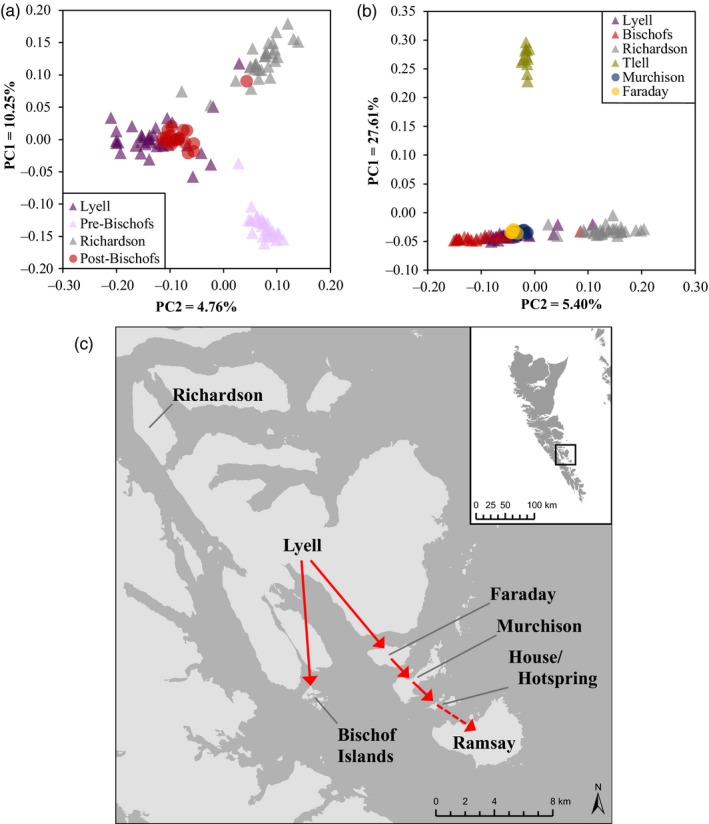
Projected principle component analysis (PCA) to identify the source of recent brown rat invasion (*Rattus norvegicus*) on (a) the Bischofs Islands (*n* = 21) and (b) Faraday (*n* = 6) and Murchison Island (*n* = 10). For all analyses, the parameter space was first defined using only samples from reference populations (triangles), and then, unknown samples were projected onto this space to identify their genetic origin (circles). For (a), *n* = 28 samples were collected prior to a failed eradication attempt on the Bischof Islands (2011) to evaluate bait failure as a cause of the eradication failure. All analyses were performed using the smartpca function from the software package EIGENSOFT v7.2.1 (Galinsky et al., 2016; Patterson et al., 2006; Price et al., 2006). Probable invasion routes are shown on (c) with solid red arrows, and the dashed arrow indicates potential future invasion

The post‐eradication population on the Bischof Islands clustered closely with the Lyell Island population using the projected PCA, though one sample did cluster with the Richardson Island population (Figure 4a). The same pattern was seen using the assignment implemented in GeneClass2.0 (Table S9). Across all analyses, a pre‐eradication origin was not supported, falsifying the survivor hypothesis.

Both Faraday Island and Murchison Island clustered with Lyell Island using the projected PCA (Figure 4b). The population assignment test implemented in GeneClass2.0 also identified a Lyell Island origin (Table S10). There was low support for a Bischof Islands origin, and no support for a Tlell (Graham Island) origin.

## 4 DISCUSSION

Managing invasive species is a global problem (Buckley, 2008; Hobbs et al., 2006). In the case of islands, eradication is the primary tool used for managing invasive species, as the impacts on native species from these invaders can be particularly severe (Glen et al., 2013; Sax & Gaines, 2008; Simberloff, Genovesi, Pyšek, & Campbell, 2011). The need for increased research when informing eradications, especially in terms of defining eradication units, has been highlighted as a means to maximize success (Buckley, 2008). Genetic data can be particularly informative in this context, providing insights on population connectivity to assist managers in defining eradication units (Abdelkrim et al., 2007; Dawson et al., 2015; Robertson & Gemmell, 2004; Russell et al., 2010).

Here, we paired archipelago‐wide sampling of brown and black rats with genotypic data at 27,686 and 10,770 SNPs, respectively, to reconstruct patterns of population connectivity and infer levels/direction of gene flow among invasive rat populations in Haida Gwaii. We found that proximate populations, for the most part, were more related than those that were more distant. Additionally, populations on the larger islands, namely Kunghit Island and Lyell Island, appeared to have greater connectivity with neighbouring populations than some of the smaller islands. Both of these observations are consistent with predictions from island biogeography theory, which states that larger, more proximate islands will share higher levels of migrants than smaller, more distant islands (MacArthur & Wilson, 1967).

### 4.1 Brown rat population connectivity

We detected three regional clusters among brown rat populations within the archipelago (Figure 2; Figure S4). For the brown rats, the northern population in Tlell (Graham Island) formed its own cluster and showed low levels of genetic diversity (Table 1, Figure 2). This population is extremely isolated from all other brown rat populations within Haida Gwaii, likely leading to its substantial population differentiation (*θ* > 0.55 in all pairwise comparisons). Lyell and its surrounding islands formed a centrally located cluster with some differentiation among island populations detected. We found low levels of differentiation among these islands, suggesting that there is some gene flow. Interestingly, Kunga Island was strongly differentiated from even the proximate Tanu Island (~1 km apart; pairwise *θ* = 0.151) and represented a unique genetic cluster across all analyses, despite the fact that significant migration rates have been recorded between brown rat populations at this distance over open ocean in the Falkland Islands (Tabak, Poncet, Passfield, Carling, & Martinez del Rio, 2015). However, Tabak, Poncet, Passfield, and del Rio (2015) did note that 97% of islands >1 km from an existing rat population remained rat free and suggested that this distance may be a threshold for brown rat dispersal over water. As such, continued introduction to Kunga Island is improbable, and the population was likely established during a single invasion event.

The southern cluster of brown rats consisted of Kunghit Island and Ellen Island. The pair was significantly differentiated, and Ellen Island consistently formed a distinct genetic cluster across analyses. This finding was also surprising, as Ellen is only ~130 m from Kunghit Island. The nearest rat population that was sampled was ~1 km away, with ~850 m of that distance over land; brown rats have been shown to have significant gene flow between populations >10 km apart, and all sample sites along the north‐west side of Kunghit Island exhibited little to no differentiation, indicating that the physical geography is not acting as a barrier. One possible explanation for the divergence between the two islands could be due to a strong ocean current, which has been shown to inhibit gene flow over such short distances (Savidge et al., 2012). However, we did identify two first‐generation migrants on Ellen Island originating from NW‐Kunghit Island, so further investigation is needed to fully understand the dynamics between these two populations.

In addition to differentiation between Ellen Island and Kunghit Island, we also detected moderate levels of divergence among the north‐west and eastern Kunghit Island populations. This divergence may be due to the topography of the Kunghit Island shoreline. While the east and west coastlines of the island are only separated by a Euclidean distance of approximately 10 km, brown rats rarely venture further than 1 km from the shoreline, so movement through the island interior is unlikely (Pye & Bonner, 1980). If the interior is acting as a barrier to dispersal, rats would have to migrate along the shoreline, which greatly increases the distance between populations. We recovered a significant IBD pattern using shoreline distance as a predictor; however, we also found a similar pattern of IBD using straight Euclidean distance, albeit with a marginally smaller effect. A more explicit landscape genetic analysis may better describe which environmental factors are affecting gene flow on Kunghit Island (Manel, Schwartz, Luikart, & Taberlet, 2003; McRae, 2006).

### 4.2 Black rat population connectivity and interspecific interactions with brown rats

Black rats also segregated into northern, central and southern clusters (Figure 3). The northern cluster was formed by populations located on Graham Island and a single population from the town of Sandspit, BC, on Moresby Island. The northern and southern sample sites were substantially differentiated from each other (pairwise *θ* > 0.142) likely due to geographic isolation, as these populations are well beyond the dispersal capacity for rats (>50 km apart) (Tabak, Poncet, Passfield, Carling, et al., 2015). Though rats sampled in Sandspit were more genetically similar to this northern cluster than the other clusters, they were still discrete from the Graham Island populations. The ocean distance is substantial between Graham Island and Moresby Island across Alliford Bay (>3 km) and poses a substantial barrier to gene flow. A regular ferry passes between the two islands and is likely the main source of population connectivity between the Graham Island and Sandspit populations. There is also regular local boat traffic between the two islands. An alternate route of dispersal between these two islands could be across Skidegate Channel (<100 m across); nonetheless, the strong population structure we observed indicates minimal connectivity even with this potential path.

The central cluster of black rats contained Lyell and surrounding islands as well as Huxley Island. In general, we found more population differentiation among island populations in this cluster than we saw among brown rat populations (Tables S5 and S7). These increases in differentiation likely arise from differences in body size and dispersal ability. Brown rats are larger bodied than black rats, which allow them to be better adapted to colder temperatures, and thus, more efficient dispersers over ocean waters (Harper, Dickinson, & Seddon, 2005). In fact, among island rat populations in the Mediterranean and New Zealand, ocean distances from 2 m to 70,000 m were significantly negatively correlated with black rat presence on islands, but there was no correlation with brown rat presence (Ruffino et al., 2009; Russell & Clout, 2004). This difference in dispersal ability can be further illustrated by the populations on Faraday Island, Murchison Island and the Bischof Islands. Both Faraday Island and Murchison Island had historical black rat populations, which were successfully eradicated in 2013 with no subsequent detection. However, these islands have been recently invaded by brown rats, most likely from the neighbouring Lyell Island (Figure 4; Tables S9 and S10). In this case, a difference in the dispersal ability between species could explain why brown rats invaded Faraday Island, and subsequently, Murchison Island, but the black rats have not re‐invaded. Furthermore, we have also shown that brown rats have re‐invaded the Bischof Islands following a 2011 eradication, which is approximately the same distance from Lyell Island (~550 m) as is Faraday Island (~700 m); black rats have never invaded the Bischof Islands though the nearest Lyell Island population is just as proximate (Burles, 2006). In addition to differences in dispersal ability, there has been recent anecdotal evidence of increases in brown rat population size on Lyell Island (R. Irvine, pers. comm.), which could be driving these rats to expand into less competitive territories (Matthysen, 2005). Once established, the brown rats can then outcompete any new invaders (*e.g.* black rats) by rapidly expanding their population numbers; in fact, the eradication of black rats on Faraday Island and Murchison Island may have even facilitated the brown rat invasion by removing any source of competition (Fraser, Banks, & Waters, 2015; Russell et al., 2010) or it simply could be due to the increased dispersal abilities of the brown rats and their increased dominance through time on adjacent Lyell Island. Additionally, historical presence of invasive rats can leave systems more prone to future invasions (Banks, Byrom, Pech, & Dickman, 2018). These interspecific interactions must be considered when planning future eradications to ensure their success.

The southern cluster of black rats consisted of only a single population from Kunghit Island. Kunghit Island historically supported a large black rat population (Bertram & Nagorsen, 1995). Brown rats invaded Kunghit Island later than the black rats, and since their arrival, black rat populations have substantially decreased. This pattern of black rat displacement by brown rats has been recorded in many systems around the world and also likely stems from the larger body size of brown rats (Atkinson, 1985; Bertram & Nagorsen, 1995; Gaston et al., 2008), a pattern observed in other small mammals (Brannon, 2000; Fox & Kirkland, 1992; Harper et al., 2005). Moreover, brown rats are almost exclusively terrestrial animals and will come into more frequent contact with seabird nests and burrows than the semi‐arboreal black rats, allowing them to be the stronger competitors for the seabird‐associated food supply (Thorsen, Shorten, Lucking, & Lucking, 2000).

### 4.3 Source(s) of recent brown rat invasions

We were able to confidently identify the source of the current Bischof Islands brown rat population as well as the invasions to Faraday Island and Murchison Island. In the case of the Bischof Islands, brown rats re‐invaded from Lyell Island and the current population were not founded by eradication survivors. Because of the short distance separating these two islands (~550 m), dispersal from Lyell Island to the Bischof Islands likely occurred without human intervention (*i.e.* brown rats swam across rather than commensally spread as stowaways on vessels). Floating across on naturals rafts of debris, such as logs with roots, is another potential vector of unaided dispersal, especially during high tides (Spennemann, 1997). Furthermore, identification of the source as re‐invaders rather than survivors indicates that the eradication methodology was effective and does not require refining to ensure complete removal of brown rats on the islands; instead, our results highlight the need for enhanced biosecurity measures.

Lyell Island was also the source population for the recent invasion onto Faraday Island and Murchison Island. From there, brown rats have now spread to House and Hotspring Islands, which has never had invasive rats of either species (C. Bergman, pers. comm., R. Irvine, pers. comm.). These invasive rat populations represent a significant biosecurity threat. Ramsay Island is situated 900–1000 m from Hotspring Island and hosts the most ecologically significant seabird colony in the Gwaii Haanas National Park (Harfenist, 2003). It also has never had a rat population, and an invasion could lead to devastating impacts on the resident seabirds.

### 4.4 Management implications

Management of highly fecund species, such as brown and black rats, can be acutely challenging, particularly with species with density‐dependent population growth rates (Moe, Stenseth, & Smith, 2002; Pardini, Drake, Chase, & Knight, 2009; Zipkin et al., 2009, 2008). Both brown and black rats have density‐dependent growth rates and recover rapidly following severe reductions in population size (Efford, Fitzgerald, Karl, & Berben, 2006; Emlen et al., 1948). Management through exclusion systems and permanent trap fixtures, such as trap‐crop and trap‐barrier systems, can mitigate the damages caused by rats on commercial crops (Singleton, Sudarmaji, & Suriapermana, 1998; Wang, Li, Li, & Guo, 2017); despite the success of such methods in agricultural settings, implementation of these methods in natural systems is an impractical solution. Long‐term poison programmes aimed at reducing rather than removing invasive rat populations are ineffective due to an increase in reproduction at low densities as well as the evolution of bait resistance or neophobia (Damin‐Pernik et al., 2017; Parsons, Banks, Deutsch, Corrigan, & Munshi‐South, 2017; Takács et al., 2016). In most cases, complete removal through eradication is the only viable method for managing invasive rat populations (Stenseth, Leirs, Mercelis, & Mwanjabe, 2002); for islands greater than 5 ha in area, the use of anticoagulant toxicants (*e.g.* brodifacoum) is currently the only effective method of eradication (Campbell et al., 2015; Leitschuh et al., 2018). While these techniques are robust in their effectiveness, there are some inherent flaws. Foremost, rodenticides are designed to be palatable to rodents in general and are not species‐specific (Campbell et al., 2015). As such, the use of rodenticides often results in mortality of nontarget organisms; within Haida Gwaii, mortality from consumption of brodifacoum‐laced pellets has been reported in dusky shrew (*Sorex monticolus*) (Taylor, Kaiser, & Drever, 2000). Additionally, mortality has been observed in common ravens (*Corvus corax*) after consumption of poison bait and consumption of poisoned rat carcasses, and brodifacoum residue was detected in blood samples from bald eagles (Howald, Mineau, Elliott, & Cheng, 1999). A potential, nonlethal alternative to rodenticides for managing invasive rat populations is gene‐drive technologies (Esvelt, Smidler, Catteruccia, & Church, 2014; Harvey‐Samuel, Ant, & Alphey, 2017); however, this research is still primarily theoretical, and much more work is needed before application in natural systems (see Leitschuh et al. (2018) and Moro, Byrne, Kennedy, Campbell, and Tizard (2018) for a thorough review). In the meantime, invasive rats still require management, highlighting the need for effective and targeted eradication efforts.

We identified several genetically isolated islands that would be suitable targets for rat eradication. The Kunga Island population was significantly isolated from all other populations and is a strong candidate for eradication, as it presents a low risk of natural reinvasion. The several smaller islets surrounding Kunga Island, such as Titul Island, should be eradicated as a single unit to prevent reinvasion to Kunga Island from these populations. Ellen Island appeared to be isolated as well and may represent a suitable candidate for eradication, though a more thorough understanding of the barriers to migration between Ellen Island and Kunghit Island is needed before action should be taken. The Shuttle Island and Huxley Island populations were identified as discrete genetic units using model‐based approaches (Figure 3c) and were moderately and significantly differentiated from all other populations (Table S7), yet PCA grouped these populations into a single unit (Figure S5B). Regardless, these islands could be eradication candidates, especially Huxley Island considering its relative isolation, though the risk of reinvasion of Shuttle Island from Lyell Island should be explicitly considered. Kunghit Island appeared to be sufficiently isolated from the north‐central populations and could also be considered as a suitable candidate for eradication. Surrounding islets should also be examined for rat populations, and these islands would need to be simultaneously eradicated to prevent reinvasion.

Additional recommendations for eradication units include Tanu Island and Richardson Island, as well as Faraday Island and Murchison Island, though these islands remain highly connected to Lyell Island, so reinvasion is probable. To successfully remove brown rats from these islands, the Lyell Island population needs to be removed or strongly controlled along the southern shore to prevent eradication failure via reinvasion; an eradication on this scale, however, would be ambitious. Refinement of methods has led to eradications on increasingly larger target areas; for example, South Georgia Island (~390,000 ha) was recently declared rat‐free following a multi‐year eradication effort (Piertney et al., 2016; Russell & Broome, 2016). Still, a Lyell Island eradication (~17,000 ha) would represent one of the largest on record (Glen et al., 2013; Russell & Broome, 2016; Springer, 2016; Towns & Broome, 2003). Though the reinvasion risk from Lyell Island remains high, eradication of brown rats from Faraday Island and Murchison Island should be viewed as a priority to protect the Ramsay Island seabird populations. Parks Canada carried out a rapid response eradication to remove brown rats from House and Hotspring Islands in November 2018 to prevent further spread, but the islands remain at risk to reinvasion.

While the term “eradication unit” inherently refers to a genetically isolated population or cluster of populations within a single species (Robertson & Gemmell, 2004), in many instances, multiple invasive species must be considered when planning effective eradications (Glen et al., 2013; Robertson & Gemmell, 2004). In the case of Haida Gwaii, brown and black rats co‐occur on five islands (Figure 1); black rats also historically occurred on Faraday and Murchison Islands, which currently host a population of brown rats. While our recommendations concerning single‐species eradication units do not change with respect to this system, future management efforts could consider the value of joint eradications when prioritizing island(s) for eradication.

### 4.5 Future directions

Here, we have provided a clear framework highlighting the utility of genomic analyses to managing invasive species. Future work could use these approaches for better understanding the mechanisms behind invasive species establishment and spread (Facon et al., 2006); in Haida Gwaii, in particular, the presence of brown and black rats provides an opportunity to take a multi‐species approach to these questions, including more explicit considerations of ecological differences among islands. Similarly, a more in‐depth examination of environmental factors affecting gene flow (*e.g.* analysis for isolation by resistance) could be used to address these same basic questions, while also adding a layer of information for planning future eradications (McRae, 2006; Piertney et al., 2016; Russell & Broome, 2016; Shah & McRae, 2008). Since levels of genetic diversity and/or behavioural flexibility can be correlated with invasion success (Cristescu, 2015; West‐Eberhard, 2003), these data could further contribute to an explicit test of the adaptive flexibility hypothesis (Wright, Eberhard, Hobson, Avery, & Russello, 2010) that predicts how the extent and direction of diversity changes at different stages of invasion. Overall, our results highlight the importance of targeted research prior to conducting invasive species eradications, both to decrease the risk of reinvasions and to prioritize recovery of at‐risk species. Invasive rats occupy the majority of oceanic islands around the world; the population genomic approach we demonstrated here could be used as a framework for guiding management of invasive rats in other island systems.

## Data Availability

All Illumina raw reads are available from the NCBI sequence read archive (BioProject ID: PRJNA592350). SNP genotypic data are deposited in DRYAD (https://doi.org/10.5061/dryad.7m0cfxpq9).
