# Supplementary material for: Rattus population genomics across the Haida Gwaii archipelago provides a framework for guiding invasive species management
Source: Evol Appl. 2020 Jan 8;13(5):889–904. doi: 10.1111/eva.12907 (PMC7232760; doi:10.1111/eva.12907)
Supplement: Supplementary file 1 [file EVA-13-889-s001.docx]

# Supplemental Tables and Figures

Table S1. Sequencing results for *n*=7 ddRAD libraries. Each library was constructed with *n*=96 individuals of black and brown rats (*Rattus rattus*, *R. norvegicus*, respectively). Libraries were sequenced on the Illumina Hi-Seq 2500 PE125 platform.

| Library | Number of reads | Number of bases | Average quality |
| --- | --- | --- | --- |
| 1 | 217,226,502 | 54,306,625,500 | 34.5 |
| 2 | 236,254,061 | 59,063,515,250 | 35 |
| 3 | 257,468,458 | 64,367,114,500 | 34 |
| 4 | 256,098,451 | 64,024,612,750 | 34 |
| 5 | 255,569,370 | 63,892,342,500 | 34 |
| 6 | 254,221,630 | 63,552,907,500 | 34 |
| 7 | 224,663,074 | 56,165,768,500 | 34 |

Figure S1. Posterior probabilities (log_10_ transformed) for *n*=673 brown (*Rattus norvegicus*) and black rats (*R. rattus*) collected in Haida Gwaii, BC for species identification. The diagonal separates assignment to species, and the dashed lines indicate 90% assignment to one species.

Table S2. Sensitivity analysis summary statistics for the brown rat (*Rattus norvegicus*) dataset (*n*=318 with replicated samples).

| r | min_maf | *N* | *N_SNP_* | Mean Depth | Mean Miss. (%) |
| --- | --- | --- | --- | --- | --- |
| 0.70 | 0.01 | 268 | 64733 | 11.95 | 11.07 |
|  | 0.02 | 272 | 55483 | 12.73 | 10.69 |
|  | 0.03 | 277 | 49563 | 13.27 | 10.44 |
|  | 0.04 | 280 | 43615 | 14.10 | 10.06 |
|  | 0.05 | 285 | 37342 | 14.75 | 10.14 |
| 0.80* | 0.01 | 282 | 41774 | 14.87 | 6.26 |
|  | 0.02 | 285 | 36844 | 15.59 | 6.11 |
|  | 0.03 | 288 | 33583 | 16.10 | 5.97 |
|  | 0.04 | 291 | 30239 | 16.85 | 5.79 |
|  | 0.05* | 294 | 25964 | 17.64 | 5.87 |
| 0.90 | 0.01 | 295 | 21011 | 21.09 | 2.24 |
|  | 0.02 | 298 | 19299 | 21.53 | 2.23 |
|  | 0.03 | 300 | 18234 | 21.75 | 2.22 |
|  | 0.04 | 300 | 17125 | 22.28 | 2.17 |
|  | 0.05 | 301 | 14764 | 23.23 | 2.19 |
| 0.95 | 0.01 | 304 | 11417 | 25.91 | 0.85 |
|  | 0.02 | 305 | 10728 | 26.09 | 0.85 |
|  | 0.03 | 305 | 10252 | 26.19 | 0.86 |
|  | 0.04 | 305 | 9780 | 26.42 | 0.86 |
|  | 0.05 | 305 | 8360 | 27.26 | 0.90 |
| r = proportion of genotyped individuals to call a SNP  min_maf = minimum minor allele frequency  *N* = number of individuals with ≥6x mean depth of coverage  *N_SNP_* = number of SNPs  Mean Depth = mean depth of coverage  Mean Miss. = mean missingness per individual  (*) denotes optimal parameter selection | | | | | |

Table S3. Sensitivity analysis summary statistics for the black rat (*Rattus rattus*) dataset (*n*=311 with replicated samples).

| r | min_maf | *N* | *N_SNP_* | Mean Depth | Mean Miss. (%) |
| --- | --- | --- | --- | --- | --- |
| 0.70 | 0.01 | 190 | 115963 | 12.22 | 7.42 |
|  | 0.02 | 204 | 88480 | 13.42 | 7.19 |
|  | 0.03 | 223 | 61871 | 14.55 | 7.51 |
|  | 0.04 | 227 | 44777 | 14.83 | 7.84 |
|  | 0.05 | 230 | 35550 | 14.95 | 8.07 |
| 0.80* | 0.01 | 230 | 62483 | 15.56 | 4.31 |
|  | 0.02 | 236 | 52906 | 16.33 | 4.24 |
|  | 0.03 | 237 | 38892 | 17.77 | 4.12 |
|  | 0.04 | 242 | 27007 | 18.10 | 4.37 |
|  | 0.05* | 245 | 20814 | 18.30 | 4.51 |
| 0.90 | 0.01 | 260 | 22056 | 21.79 | 1.98 |
|  | 0.02 | 260 | 19694 | 22.08 | 2.03 |
|  | 0.03 | 260 | 14970 | 22.41 | 1.98 |
|  | 0.04 | 260 | 9270 | 22.58 | 1.87 |
|  | 0.05 | 263 | 6290 | 22.86 | 1.82 |
| 0.95 | 0.01 | 280 | 1524 | 27.18 | 0.57 |
|  | 0.02 | 280 | 1193 | 27.34 | 0.63 |
|  | 0.03 | 281 | 928 | 27.30 | 0.64 |
|  | 0.04 | 280 | 533 | 27.28 | 0.60 |
|  | 0.05 | 280 | 352 | 27.61 | 0.53 |
| r = proportion of genotyped individuals to call a SNP  min_maf = minimum minor allele frequency  *N* = number of individuals with ≥6x mean depth of coverage  *N_SNP_* = number of SNPs  Mean Depth = mean depth of coverage  Mean Miss. = mean missingness per individual  (*) denotes optimal parameter selection | | | | | |

Table S4. Summary statistics for the filtered brown rat and black rat datasets. Mean depth and missingness were calculated using VCFtools v0.1.15 (Danecek et al., 2011). Mean genotyping error was calculated as percent discordance in genotypes between replicate individuals and was calculated both with and among sequencing libraries.

|  |  |  |  |  |  | Genotyping Error (%) | |
| --- | --- | --- | --- | --- | --- | --- | --- |
| Dataset | *N* | *N*_UNIQUE_ | *N*_SNP_ | Mean Depth | Mean Miss. (%) | Within | Among |
| Brown | 297 | 283 | 27,686 | 16.1x | 7.7 | 2.00 | 2.57 |
| Black | 242 | 238 | 10,770 | 21.4x | 2.5 | 4.17 | 2.50 |
| *N* = number of total individuals (with replicates)  *N*_UNIQUE_ = number of unique individuals (no replicates)  *N*_SNP_ = number of SNPs  Mean Depth = mean depth of coverage across all individuals  Mean Miss. = mean percent missing genotypes across all individuals | | | | | |  |  |

Table S5. Matrix of pairwise population differentiation (Weir & Cockerham θ (1984)) for *n*=283 brown rats (*Rattus norvegicus*) collected across Haida Gwaii, BC. Significance is indicated above the diagonal and calculated over 1000 permutations after Bonferroni correction. Abbreviations are defined as follows: (TLE) Tlell; (PRE) Pre-Bischofs; (POST) Post-Bischofs; (LYL) Lyell; (RIC) Richardson; (TNU) Tanu; (KGA) Kunga; (FAR) Faraday; (MUR) Murchison; (NGKT) NW-Kunghit; (EKGT) E-Kunghit; and (ELI) Ellen.

| Population | TLE | PRE | POST | LYL | RIC | TNU | KGA | FAR | MUR | NKGT | EKGT | ELI |
| --- | --- | --- | --- | --- | --- | --- | --- | --- | --- | --- | --- | --- |
| Tlell | - | ** | ** | ** | ** | ** | ** | ** | ** | ** | ** | ** |
| Pre-Bischofs | 0.752 | - | ** | ** | ** | ** | ** | ** | ** | ** | ** | ** |
| Post-Bischofs | 0.679 | 0.208 | - | ** | ** | ** | ** | ** | ** | ** | ** | ** |
| Lyell | 0.620 | 0.138 | 0.065 | - | ** | ** | ** | ** | ** | ** | ** | ** |
| Richardson | 0.601 | 0.225 | 0.133 | 0.086 | - | ** | ** | ** | ** | ** | ** | ** |
| Tanu | 0.597 | 0.229 | 0.136 | 0.086 | 0.073 | - | ** | ** | ** | ** | ** | ** |
| Kunga | 0.673 | 0.342 | 0.249 | 0.201 | 0.189 | 0.151 | - | ** | ** | ** | ** | ** |
| Faraday | 0.759 | 0.367 | 0.239 | 0.164 | 0.219 | 0.217 | 0.359 | - | ** | ** | ** | ** |
| Murchison | 0.734 | 0.319 | 0.206 | 0.128 | 0.186 | 0.193 | 0.323 | 0.073 | - | ** | ** | ** |
| NW-Kunghit | 0.574 | 0.316 | 0.275 | 0.246 | 0.271 | 0.271 | 0.354 | 0.314 | 0.303 | - | ** | ** |
| E-Kunghit | 0.607 | 0.393 | 0.346 | 0.315 | 0.335 | 0.334 | 0.420 | 0.376 | 0.367 | 0.080 | - | ** |
| Ellen | 0.667 | 0.472 | 0.415 | 0.378 | 0.394 | 0.393 | 0.482 | 0.454 | 0.445 | 0.138 | 0.198 | - |
| (*) *p*<0.05; (**) *p*<0.01 | | | | | | | | | | | | |

Figure S2. Principle component analyses of brown rats (*Rattus norvegicus*) collected from a) centrally located islands (*n*=187) and b) southerly located islands (*n*=82) within the Haida Gwaii archipelago. The “NW Kunghit”, “E Kunghit”, and “Luxana Bay” populations were all collected from Kunghit island.

Figure S3. Mean cross-entropy estimates for *k*=1-20 from a Bayesian clustering analysis implemented by the R-package *LEA* v2.6.0 (Frichot & François, 2015) for *n*=283 brown rats (*Rattus norvegicus*) collected in Haida Gwaii, BC. Cross entropy estimates were averaged over *n*=10 iterations for each value of *k*. The location of “elbow” (indicated by the dashed line) identified an optimal *k*=9.

Figure S4. Mean (top) and individual (bottom) ancestry coefficients for *n*=283 brown rats (*Rattus norvegicus*) sampled across Haida Gwaii, BC. Ancestry coefficients were calculated using the R-package *LEA* v2.4.0 (Frichot & François, 2015); mean ancestry coefficients are shown an at an optimal number of genetic clusters *k*=9. (*) indicates pre-eradication samples (*n*=28) collected on the Bischof Islands. Map labels represent sample sites within an island; those sites that share a superscript were grouped into a single population. Sample site definitions are as follows: (TI) Titul Island; (KI) Kunga Island; (RP) Richardson Point – Lyell Island; (SP) Sedgewick Point – Lyell Island; (FP) Faraday Passage – Lyell Island; (HP) Hornby Point – Kunghit Island; (AP) Arnold Point – Kunghit Island; (BP) Bowles Point – Kunghit Island; (GB) Graham Bay – Kunghit Island; (LB) Luxana Bay – Kunghit Island; (KB) Keeweenah Bay – Kunghit Island; (MI) Marshall Island; and (RI) Rainy Islands.

Table S6. Mean directional migration rates for *n*=283 brown rats (*Rattus norvegicus*) sampled within Haida Gwaii, BC. Source populations are arranged in rows and sink populations are arranged in columns. Migration rates were averaged across five iterations and calculated using the software BayesAss3-SNPs v1.1.0 (Mussmann, Douglas, Chafin, & Douglas, in press; Wilson & Rannala, 2003). 95% credible sets were calculated as mean standard deviation * 1.96 (parentheses); migration rates were deemed significant if the credible set did not include zero (shaded grey). Bolded values along the diagonal represent the fraction of individuals that remain in a population. Abbreviation definitions are as follows: (POST) post-Bischofs; (PRE) pre-Bischofs; (ELI) Ellen; (FAR) Faraday; (TLE) Tlell; (NKGT) NW Kunghit; (EKGT) E Kunghit; (KGA) Kunga; (LYL) Lyell; (MUR) Murchison; (RIC) Richardson; and (TNU) Tanu.

| Source:SINK | ELI | FAR | KGA | LYL | MUR | NKGT | POST | PRE | RIC | TNU | TLE | EKGT |
| --- | --- | --- | --- | --- | --- | --- | --- | --- | --- | --- | --- | --- |
| Ellen | **0.860** | 0.011 | 0.011 | 0.011 | 0.011 | 0.032 | 0.011 | 0.011 | 0.011 | 0.011 | 0.011 | 0.011 |
|  | **(0.057)** | (0.02) | (0.02) | (0.02) | (0.02) | (0.034) | (0.02) | (0.02) | (0.02) | (0.02) | (0.02) | (0.02) |
| Faraday | 0.019 | **0.796** | 0.019 | 0.019 | 0.019 | 0.018 | 0.019 | 0.019 | 0.018 | 0.018 | 0.019 | 0.019 |
|  | (0.034) | **(0.073)** | (0.034) | (0.035) | (0.035) | (0.034) | (0.034) | (0.034) | (0.034) | (0.034) | (0.035) | (0.034) |
| Kunga | 0.008 | 0.008 | **0.917** | 0.008 | 0.008 | 0.008 | 0.008 | 0.008 | 0.008 | 0.008 | 0.008 | 0.007 |
|  | (0.015) | (0.015) | **(0.042)** | (0.014) | (0.015) | (0.015) | (0.015) | (0.014) | (0.014) | (0.015) | (0.015) | (0.014) |
| Lyell | 0.007 | 0.007 | 0.007 | **0.899** | 0.007 | 0.007 | 0.007 | 0.007 | 0.022 | 0.014 | 0.007 | 0.007 |
|  | (0.014) | (0.014) | (0.014) | **(0.044)** | (0.014) | (0.014) | (0.014) | (0.014) | (0.024) | (0.019) | (0.014) | (0.014) |
| Murchison | 0.015 | 0.030 | 0.015 | 0.015 | **0.818** | 0.015 | 0.015 | 0.015 | 0.015 | 0.015 | 0.015 | 0.015 |
|  | (0.028) | (0.039) | (0.029) | (0.028) | **(0.068)** | (0.028) | (0.028) | (0.029) | (0.028) | (0.028) | (0.028) | (0.028) |
| NW-Kunghit | 0.007 | 0.007 | 0.021 | 0.007 | 0.007 | **0.910** | 0.007 | 0.007 | 0.007 | 0.007 | 0.007 | 0.007 |
|  | (0.013) | (0.013) | (0.023) | (0.013) | (0.013) | **(0.041)** | (0.013) | (0.013) | (0.013) | (0.013) | (0.013) | (0.013) |
| Post-Bischofs | 0.010 | 0.010 | 0.010 | **0.039** | 0.010 | 0.010 | **0.850** | 0.010 | 0.020 | 0.010 | 0.010 | 0.010 |
|  | (0.019) | (0.019) | (0.019) | **(0.037)** | (0.019) | (0.019) | **(0.056)** | (0.019) | (0.027) | (0.019) | (0.019) | (0.019) |
| Pre-Bischofs | 0.008 | 0.008 | 0.008 | 0.017 | 0.008 | 0.008 | 0.008 | **0.900** | 0.008 | 0.008 | 0.008 | 0.008 |
|  | (0.016) | (0.016) | (0.016) | (0.022) | (0.016) | (0.016) | (0.016) | **(0.047)** | (0.016) | (0.016) | (0.016) | (0.016) |
| Richardson | 0.008 | 0.008 | 0.008 | 0.017 | 0.008 | 0.008 | 0.008 | 0.008 | **0.897** | 0.014 | 0.008 | 0.008 |
|  | (0.015) | (0.015) | (0.015) | (0.022) | (0.015) | (0.015) | (0.015) | (0.015) | **(0.046)** | (0.02) | (0.015) | (0.015) |
| Tanu | 0.009 | 0.009 | 0.009 | 0.009 | 0.009 | 0.009 | 0.009 | 0.009 | 0.026 | **0.887** | 0.009 | 0.009 |
|  | (0.017) | (0.017) | (0.017) | (0.017) | (0.017) | (0.017) | (0.017) | (0.017) | (0.028) | **(0.05)** | (0.017) | (0.017) |
| Tlell | 0.013 | 0.013 | 0.013 | 0.013 | 0.013 | 0.013 | 0.013 | 0.013 | 0.013 | 0.013 | **0.859** | 0.013 |
|  | (0.024) | (0.024) | (0.024) | (0.024) | (0.024) | (0.024) | (0.024) | (0.024) | (0.024) | (0.024) | **(0.062)** | (0.024) |
| E-Kunghit | 0.009 | 0.009 | 0.009 | 0.009 | 0.009 | **0.031** | 0.009 | 0.009 | 0.009 | 0.008 | 0.009 | **0.883** |
|  | (0.016) | (0.016) | (0.016) | (0.016) | (0.016) | **(0.031)** | (0.016) | (0.016) | (0.016) | (0.016) | (0.016) | **(0.05)** |

Table S7. Matrix of pairwise population differentiation (Weir & Cockerham θ (1984)) for *n*=238 black rats (*Rattus rattus*) collected across Haida Gwaii, BC. Significance is indicated above the diagonal and calculated over 1000 permutations after Bonferroni correction. Abbreviations are defined as follows: (NGRM) Graham-N; (SGRM) Graham-S; (SAND) Sandspit; (FAR) Faraday; (MUR) Murchison; (LFP) Lyell-FP; (LSW) Lyell-SW; (SHTL) Shuttle; (HUX) Huxley; and (KGT) Kunghit.

| Population | NGRM | SGRM | SAND | FAR | MUR | LFP | LSW | SHTL | HUX | KGT |
| --- | --- | --- | --- | --- | --- | --- | --- | --- | --- | --- |
| Graham-N | - | ** | ** | ** | ** | ** | ** | ** | ** | ** |
| Graham-S | 0.142 | - | ** | ** | ** | ** | ** | ** | ** | ** |
| Sandspit | 0.380 | 0.321 | - | ** | ** | ** | ** | ** | ** | ** |
| Faraday | 0.457 | 0.403 | 0.434 | - | ** | ** | ** | ** | ** | ** |
| Murchison | 0.404 | 0.353 | 0.361 | 0.176 | - | ** | ** | ** | ** | ** |
| Lyell-FP | 0.456 | 0.416 | 0.434 | 0.272 | 0.215 | - | ** | ** | ** | ** |
| Lyell-SW | 0.430 | 0.387 | 0.397 | 0.250 | 0.196 | 0.097 | - | ** | ** | ** |
| Shuttle | 0.478 | 0.431 | 0.480 | 0.380 | 0.313 | 0.286 | 0.244 | - | ** | ** |
| Huxley | 0.484 | 0.430 | 0.488 | 0.410 | 0.341 | 0.328 | 0.293 | 0.352 | - | ** |
| Kunghit | 0.468 | 0.406 | 0.511 | 0.495 | 0.398 | 0.451 | 0.407 | 0.460 | 0.463 | - |
| (*) p<0.05; (**) p<0.01 | | | | | | | | | | |

Figure S5. Principle component analyses of brown rats (*Rattus rattus*) collected from a) northerly located islands (*n*=52) and b) centrally located islands (*n* =182) within the Haida Gwaii archipelago. The “Graham-North” and “Graham-South” populations were both collected from Graham Island, and the “Lyell-FP” and “Lyell-SW” populations were collected from Lyell Island.

Figure S6. Mean cross-entropy estimates for *k*=1-20 from a Bayesian clustering analysis implemented by the R-package *LEA* v2.6.0 (Frichot & François, 2015) for *n*=238 black rats (*Rattus rattus*) collected in Haida Gwaii, BC. Cross entropy estimates were averaged over *n*=10 iterations for each value of *k*. The location of “elbow” (indicated by the dashed line) identified an optimal *k*=9.

Figure S7. Mean (top) and individual (bottom) ancestry coefficients for *n*=238 black rats (*Rattus rattus*) sampled across Haida Gwaii, BC. Ancestry coefficients were calculated using the R-package *LEA* v2.4.0 (Frichot & François, 2015); mean ancestry coefficients are shown an at an optimal number of genetic clusters *k*=9. Map labels represent sample sites within an island; those sites that share a superscript were grouped into a single population. Sample site definitions are as follows: (MS) Masset – Graham Island; (TT) Tow Town – Graham Island; (TH) Tow Hill – Graham Island; (PC) Parks Canada Dump – Graham Island; (TL) Tlell – Graham Island; (MC) Miller Creek – Graham Island; (SK) Skidegate – Graham Island; (QC) Queen Charlotte – Graham Island; (LW) Lyell Island West; (RP) Richardson Point – Lyell Island; (SP) Sedgewick Point – Lyell Island; and (FP) Faraday Passage – Lyell Island.

Table S8. Mean directional migration rates for *n*=238 black rats (*Rattus rattus*) sampled within Haida Gwaii, BC. Source populations are arranged in rows and sink populations are arranged in columns. Migration rates were averaged across five iterations and calculated using the software BayesAss3-SNPs v1.1.0 (Mussmann et al., in press; Wilson & Rannala, 2003). 95% credible sets were calculated as mean standard deviation * 1.96 (indicated in parentheses); migration rates were deemed significant if the credible set did not include zero (shaded grey). Bolded values along the diagonal represent the fraction of individuals that remain in a population. Abbreviation definitions are as follows: (FAR) Faraday; (NGRM) Graham-North; (SGRM) Graham-South; (HUX) Huxley; (KGT) Kunghit; (LFP) Lyell-FP; (LSW) Lyell-SW; (SAND) Sandspit; (MUR) Murchison; and (SHTL) Shuttle.

| Source:SINK | FAR | NGRM | SGRM | HUX | KGT | LFP | LSW | SAND | MUR | SHTL |
| --- | --- | --- | --- | --- | --- | --- | --- | --- | --- | --- |
| Faraday | **0.875** | 0.014 | 0.014 | 0.014 | 0.014 | 0.014 | 0.014 | 0.014 | 0.014 | 0.014 |
|  | **(0.063)** | (0.026) | (0.026) | (0.026) | (0.026) | (0.026) | (0.026) | (0.026) | (0.026) | (0.026) |
| Graham-N | 0.010 | **0.892** | 0.029 | 0.010 | 0.010 | 0.010 | 0.010 | 0.010 | 0.010 | 0.010 |
|  | (0.019) | **(0.052)** | (0.031) | (0.019) | (0.019) | (0.019) | (0.019) | (0.019) | (0.019) | (0.019) |
| Graham-S | 0.010 | 0.010 | **0.909** | 0.010 | 0.010 | 0.010 | 0.010 | 0.010 | 0.010 | 0.010 |
|  | (0.019) | (0.019) | **(0.050)** | (0.019) | (0.019) | (0.019) | (0.019) | (0.019) | (0.019) | (0.019) |
| Huxley | 0.013 | 0.013 | 0.013 | **0.880** | 0.013 | 0.013 | 0.013 | 0.013 | 0.013 | 0.013 |
|  | (0.025) | (0.025) | (0.025) | **(0.062)** | (0.025) | (0.025) | (0.025) | (0.025) | (0.025) | (0.025) |
| Kunghit | 0.024 | 0.024 | 0.024 | 0.024 | **0.786** | 0.024 | 0.024 | 0.024 | 0.024 | 0.024 |
|  | (0.044) | (0.043) | (0.044) | (0.043) | **(0.081)** | (0.043) | (0.043) | (0.044) | (0.044) | (0.044) |
| Lyell-FP | 0.005 | 0.006 | 0.005 | 0.005 | 0.005 | **0.948** | 0.009 | 0.005 | 0.005 | 0.005 |
|  | (0.011) | (0.011) | (0.011) | (0.011) | (0.011) | **(0.030)** | (0.013) | (0.011) | (0.010) | (0.010) |
| Lyell-SW | 0.005 | 0.005 | 0.005 | 0.005 | 0.005 | **0.052** | **0.908** | 0.005 | 0.005 | 0.005 |
|  | (0.010) | (0.010) | (0.010) | (0.010) | (0.010) | **(0.029)** | **(0.036)** | (0.010) | (0.010) | (0.010) |
| Sandspit | 0.022 | 0.022 | 0.022 | 0.022 | 0.022 | 0.022 | 0.022 | **0.800** | 0.022 | 0.022 |
|  | (0.041) | (0.041) | (0.040) | (0.041) | (0.041) | (0.041) | (0.041) | **(0.080)** | (0.041) | (0.041) |
| Murchison | 0.010 | 0.010 | 0.010 | 0.010 | 0.010 | 0.010 | 0.010 | 0.010 | **0.906** | 0.010 |
|  | (0.020) | (0.020) | (0.020) | (0.020) | (0.020) | (0.020) | (0.020) | (0.020) | **(0.051)** | (0.020) |
| Shuttle | 0.010 | 0.010 | 0.010 | 0.010 | 0.010 | 0.010 | 0.010 | 0.010 | 0.010 | **0.912** |
|  | (0.019) | (0.019) | (0.019) | (0.019) | (0.019) | (0.019) | (0.018) | (0.019) | (0.019) | **(0.049)** |

Table S9. Population assignment test for *n*=21 brown rats (*Rattus norvegicus*) collected from the Bischof Islands, Haida Gwaii following a failed eradication. Putative source populations included Lyell and Richardson Island, while the Pre-Bischofs population were samples collected prior to the failed eradication. Population assignments were carried out under the Rannala and Mountain (1997) criterion as implemented in GeneClass v2.0.h (Piry et al., 2004).

| Sample ID | Probability of Assignment | | |  | -log(likelihood) | | |
| --- | --- | --- | --- | --- | --- | --- | --- |
|  | Pre-Bischofs | Lyell | Richardson |  | Pre-Bischofs | Lyell | Richardson |
| BIA-16-001_4-1 | 0 | 100 | 0 |  | 1679 | 1181 | 1465 |
| BIA-16-002 | 0 | 100 | 0 |  | 1616 | 1151 | 1420 |
| BIA-16-003 | 0 | 100 | 0 |  | 1680 | 1170 | 1419 |
| BIA-16-004 | 0 | 100 | 0 |  | 1712 | 1161 | 1438 |
| BIA-16-005 | 0 | 100 | 0 |  | 1459 | 984 | 1227 |
| BID-16-001 | 0 | 100 | 0 |  | 1505 | 1177 | 1452 |
| BID-16-002 | 0 | 100 | 0 |  | 1505 | 1033 | 1345 |
| BIS-001 | 0 | 100 | 0 |  | 1429 | 959 | 1194 |
| BIS-002 | 0 | 100 | 0 |  | 1500 | 1015 | 1212 |
| BIS-003 | 0 | 100 | 0 |  | 1599 | 1064 | 1308 |
| BIS-004 | 0 | 100 | 0 |  | 1479 | 1021 | 1243 |
| BIS-005 | 0 | 100 | 0 |  | 1327 | 947 | 1159 |
| BIS-006_2 | 0 | 100 | 0 |  | 1622 | 1096 | 1261 |
| BIS-007 | 0 | 100 | 0 |  | 1568 | 1039 | 1326 |
| BIS-008_2 | 0 | 100 | 0 |  | 1688 | 1111 | 1300 |
| BIS-009 | 0 | 100 | 0 |  | 1605 | 1081 | 1284 |
| BIS-010_2 | 0 | 100 | 0 |  | 1688 | 1148 | 1431 |
| BIS-011 | 0 | 100 | 0 |  | 1504 | 1006 | 1257 |
| BIS-012 | 0 | 0 | 100 |  | 1785 | 1195 | 1013 |
| BIS-013 | 0 | 100 | 0 |  | 1679 | 1112 | 1356 |
| BIS-13-001 | 0 | 100 | 0 |  | 1341 | 970 | 1202 |

Table S10. Population assignment test for brown rats (*Rattus norvegicus*) collected from Faraday Island (*n*=6) and Murchison Island (*n*=10), Haida Gwaii. Putative source populations included Lyell, Richardson, and the Bischof Islands, as well as Tlell, BC. Population assignments were carried out under the Rannala and Mountain (1997) criterion as implemented in GeneClass v2.0.h (Piry et al., 2004).

|  | Probability of Assignment | | | |  | -log(likelihood) | | | |
| --- | --- | --- | --- | --- | --- | --- | --- | --- | --- |
| Sample ID | Bischofs | Tlell | Lyell | Richardson |  | Bischofs | Tlell | Lyell | Richardson |
| FAR-18-01a | 0 | 0 | 100 | 0 |  | 1380 | 4585 | 1147 | 1385 |
| FAR-18-02a | 0 | 0 | 100 | 0 |  | 1322 | 4519 | 1135 | 1411 |
| FAR-18-03a_2 | 0 | 0 | 100 | 0 |  | 1382 | 4613 | 1189 | 1465 |
| FAR-18-04a | 0 | 0 | 100 | 0 |  | 1277 | 4452 | 1074 | 1332 |
| FAR-18-05a | 0 | 0 | 100 | 0 |  | 1333 | 4382 | 1135 | 1390 |
| FAR-18-06a | 0 | 0 | 100 | 0 |  | 1261 | 4116 | 1097 | 1332 |
| MUR-18-01a_2 | 0 | 0 | 100 | 0 |  | 1437 | 4700 | 1158 | 1395 |
| MUR-18-03a | 0 | 0 | 100 | 0 |  | 1247 | 4268 | 1079 | 1226 |
| MUR-18-04a | 0 | 0 | 100 | 0 |  | 1324 | 4243 | 1110 | 1278 |
| MUR-18-05a | 0 | 0 | 100 | 0 |  | 1342 | 4314 | 1097 | 1340 |
| MUR-18-06a | 0 | 0 | 100 | 0 |  | 1343 | 4334 | 1100 | 1362 |
| MUR-18-07a | 0 | 0 | 100 | 0 |  | 1045 | 3763 | 847 | 1044 |
| MUR-18-08a | 0 | 0 | 100 | 0 |  | 1529 | 4554 | 1248 | 1461 |
| MUR-18-09a | 0 | 0 | 100 | 0 |  | 1302 | 4494 | 1109 | 1343 |
| MUR-18-10a | 0 | 0 | 100 | 0 |  | 1043 | 4002 | 859 | 1094 |
| MUR-18-11a | 0 | 0 | 100 | 0 |  | 1533 | 4689 | 1235 | 1435 |
